# The biological functions of target genes in pan-cancers and cell lines were predicted by miR-375 microarray data from GEO database and bioinformatics

**DOI:** 10.1371/journal.pone.0206689

**Published:** 2018-10-31

**Authors:** Jiang-Hui Zeng, Xu-Zhi Liang, Hui-Hua Lan, Xu Zhu, Xiu-Yun Liang

**Affiliations:** 1 Department of ClinicaHl Laboratory, The Third Affiliated Hospital of Guangxi Medical University/Nanning Second People’s Hospital, Nanning, Guangxi Zhuang Autonomous Region, P. R. China; 2 Department of Pathology, The First Affiliated Hospital of Guangxi Medical University, Nanning, Guangxi Zhuang Autonomous Region, P. R. China; 3 Department of Clinical Laboratory, The People's Hospital of Guangxi Zhuang Autonomous Region, Nanning, Guangxi Zhuang Autonomous Region, P. R. China; University of South Alabama Mitchell Cancer Institute, UNITED STATES

## Abstract

**Background:**

MicroRNA is endogenous non-coding small RNA that negative regulate and control gene expression, and increasing evidence links microRNA to oncogenesis and the pathogenesis of cancer. The goal of this study was to explore the potential molecular mechanism of miR-375 in various cancers.

**Methods:**

MiR-375 overexpression in different tumor cell lines was probed with microarray data from Gene Expression Omnibus (GEO). The common target genes of miR-375 were obtained by Robust Rank Aggregation (RRA), and identified by miRWalk2.0 software for target gene prediction. Additionally, we directed in silico analysis including Protein-Protein Interactions (PPI) analysis, gene ontology (GO) enrichment analysis and Kyoto Encyclopedia of Genes and Genomes (KEGG) pathways annotations to provide a summary of the function of miR-375 in various carcinomas. Eventually, data was obtained from The Cancer Genome Atlas (TCGA) were utilized for a validation in 7 cancers.

**Results:**

The nine miR-375 related chips were acquired by the GEO data. The 5 down regulated genes came from 9 available microarray datasets, which overlapped with the potential target genes predicted by miRWalk2.0 software. The target genes were intensely enriched in amino acid biosynthetic and metabolic process from biological process (GO) and Cysteine and methionine metabolism (KEGG analysis). In view of these approaches, VASN, MAT2B, HERPUD1, TPAPPC6B and TAT are probably the most important miR-375 targets. In addition, miR-375 was negatively correlated with MAT2B, which was verified in 5 tumors of TCGA.

**Conclusion:**

In summary, this study based on common target genes provides an innovative perspective for exploring the molecular mechanism of miR-375 in human tumors.

## Introduction

MiRNA abnormalities are crucial for the formation and development of cancer and have a regulatory effect on the sensitivity of various cancer treatments. MiR-375 is a widely studied miRNA that has been reported to contribute to the development and progression of several malignancies [1–3]. MicroRNAs (MiRNAs) are called for its feature of small, non-coding RNA molecules that contain 19–24 nucleotides [4]. Currently, 1881 unique Homo sapiens miRNAs and 2588 mature miRNAs are recorded in miRBase v20. The number is still increasing (http://www.mirbase.org/) [5]. Although miRNAs do not encode proteins, they can be used to regulate the expression level of a target gene by base-pairing with the transcripts of the target protein-encoding gene to reduce or inhibit the target gene [6]. The target gene silencing mechanism relies on the state of the complementary sequence between the miRNA and the 3 'untranslated region (UTR) of the target massager Ribonucleic Acid (mRNA). If there is complete base-pairing homology between the miRNA and its target mRNA, the RNA interference pathway is activated, which eventually cleaves the target mRNA. However, binding is often imperfect, and often occurs, target mRNA translation will be modulated, severely interfering with mRNA expression [7]. Based on these theories, there is often a reverse association between the level of miRNA expression and its target gene. In addition, the state of complementarity between a miRNA and its target provide an opportunity to predict the potential target of a miRNA. Although some targets of miR-375 have been confirmed, there may be more potential target genes. Therefore, we extracted gene expression data from multiple in vivo malignancies, including lung, kidney, head and neck, prostate and bladder cancer, following a miR-375 mock transfection. We also attempted to provide a comprehensive overview of the potential molecular mechanisms of miR-375 in cancer and provide a new perspective for the study of miR-375.

## Material and methods

### Collection of relevant microarrays

**Fig 1:**
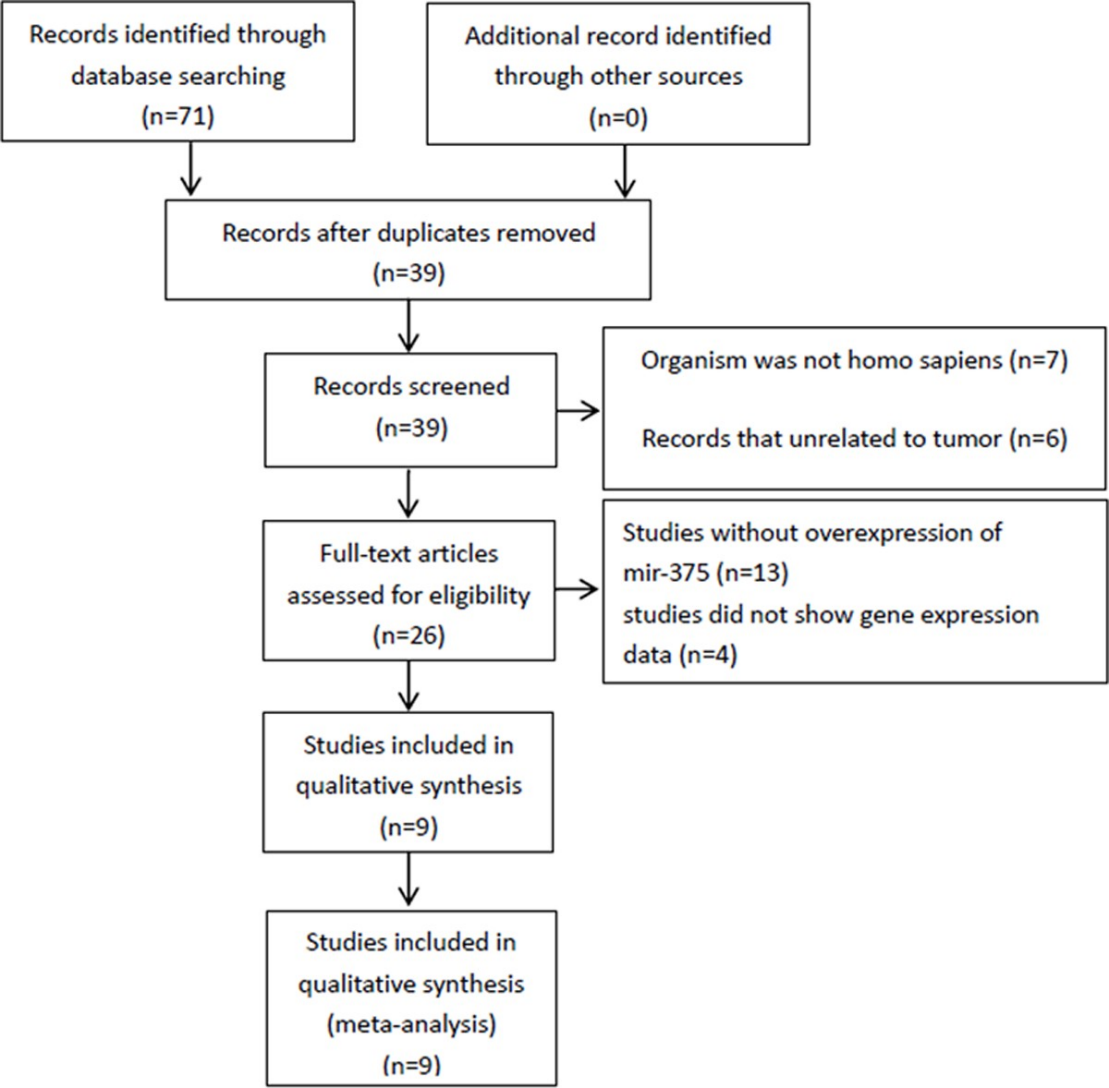
The researchers obtained 9 RNA microarray datasets of miR-375 from the GEO database.

Related microarrays are collected from Gene Expression Omnibus (GEO) database (http://www.ncbi.nlm.nih.gov/geo/). In our study, the mir-375 overexpressed microarrays related to different cancer cell lines was limited to those were published before 2017-10-25. The following retrieval models are used for retrieval, search terms were: microrna-375 OR miR-375 OR mirna-375 OR hsa-miR-375 OR MicroRNA375. In total, 71 studies were obtained and eliminate duplicate 32 items, then 13 studies were excluded because they were not homo sapiens (n = 7) or tumor diseases (n = 6). Moreover, 17 studies were rejected, 13 of which were unrelated to over expression of mir-375, and four had no gene expression data. Finally, we get nine microarray series (Fig 1). Series Matrix files and platform annotation files were obtained and parsed by Gene Expression Omnibus GEO query package of R language. All Series Matrix files, annotations, platform sets and files were performed by the GEO query package with R version 3.2.2 in Bioconducter 3.2.

### Box-plots

Box-plots were utilized for individually included GSE microarray data to show inter-group variability. We premier prepared the Box plots representation of median-centered gene expression to elucidate whether the microarray data of GSE were corresponding [8].

### Common target genes of miR-375 assessed by Robust Rank Aggregation (RRA)

RRA is a packet of R language for clustering those genes related to statistics, which implemented as a probabilistic model is robust to outlier, noise and error [9]. It uses strict scoring methods to obtain statistically related genes to keep the algorithm parameters being not disturbed.

Step 1: All chip probes are annotated with the gene name according to the annotation information provided by the platform.

Step 2: The single channel chip uses the limma package to identify the differential gene [10,11], and the ratio of cy5 to cy3 of the dual—channel chip is converted to a log2 ratio.

Step 3: the RRA model was used to evaluate the expression of the above differences. Under the standard of FDR< 0.05 and |logFC|>1, the gene of common expression trend was obtained by RRA mode in the form of scoring.

### MiR-375 target gene prediction

We used miRWalk2.0 software to predict miR-375 target genes (including RNAhybrid[12], Targetscan[13], miRDB[14], miRMap[15], miRWalk[16], Microt4[17], RNA22[18], miRanda[19], mirbridge[20], miRNAMap[21], Pictar2[22] and PITA[23])[24–26], the genes that were predicted by three or more software at the same time were considered to be its target genes.

### MiR-375 protein and protein interaction (PPI) and pathways analysis

The common low expression gene of RRA is the potential target gene of miR-375. What’s more, the protein interactions (PPI) of the possible target genes of mir-375 were constructed via a STRING (http://string-db.org/). We conducted a enrichment analysis of the GO and KEGG pathway to perceive the biological functions and pathways of the potential target genes of mir-375.

### MiR-375 expression levels in cancers based on TCGA data

We used The GDC Data Portal (https://gdc-portal.nci.nih.gov/) from The TCGA database to download the miRNA-seq and RNA-seq Data of 7 tumors. We extracted the expression data of miR-375 and target genes. Students' t-test analyzed the expression differences between the normal group and the cancer group. The correlation between mir-375 and target genes was illustrated by Pearson's correlation test.

## Results

### Nine miR-375 microarray datasets

As shown in Fig 1, we systematically review the GEO database chip series for determining our research, 9 studies were chosen for the following analysis. We obtained nine chip series from the GEO database through rigorous selection. An overview of the nine chip series are shown in Table 1.

**Table 1:** A list of individual data sets of miR-375 overexpressing from GEO dataset.

Study	GEO	Platform	Sample	Cancer type	References
	accession				
1*	GSE26032	GPL10332 (Agilent-026652 Whole Human Genome Microarray 4x44K v2)	GSM639299 (SAS)	Head and neck squamous cell carcinoma	21753766
			GSM639300 (FaDu)		
2*	GSE31566	GPL6480 (Agilent-014850 Whole Human Genome Microarray 4x44K G4112F)	GSM783360 (A549)	Lung cancer	21856745
			GSM783361 (A549)		
			GSM783362 (A549)		
			GSM783363 (A549)		
3*	GSE37119	GPL10332 (Agilent-026652 Whole Human Genome Microarray 4x44K v2)	GSM911052 (IMC-3)	Head and neck squamous cell carcinoma Esophageal squamous cell carcinoma	21922130
			GSM911060 (T.Tn)		
			GSM911061 (TE2)		
4*	GSE77790	GPL20844 (Agilent-072363 SurePrint G3 Human GE v3 8x60K Microarray 039494)	GSM2059428 (Panc-1)	Pancreatic cancer Esophageal cancer	27862697
			GSM2059429 (sw1990)		
			GSM2059430 (TE8)		
			GSM2059431 (TE9)		
5*	GSE47657	GPL13607 (Agilent-028004 SurePrint G3 Human GE 8x60K Microarray)	GSM1154167 (FaDu)	Head and neck squamous cell carcinoma	24091622
6	GSE58860	GPL10558 (Illumina HumanHT-12 V4.0 expression beadchip)	GSM1420974 (SN1)	Colon cancer	\
			GSM1420975 (SN2)		
			GSM1420980 (S375-1)		
			GSM1420981 (S375-2)		
7	GSE67742	GPL16699 (Agilent-039494 SurePrint G3 Human GE v2 8x60K Microarray 039381)	GSM1655071 (ORI)	Medullary thyroid carcinoma	27036030
			GSM1655073 (ORI)		
			GSM1655074 (ORI)		
			GSM1655075 (ORI)		
8	GSE40058	GPL6244 ([HuGene-1_0-st] Affymetrix Human Gene 1.0 ST Array)	GSM984506 (MDA-MB-231) GSM984509 (MDA-MB-231)	Breast cancer	23497265
9	GSE74213	GPL6480 (Agilent-014850 Whole Human Genome Microarray 4x44K G4112F)	GSM1914647 (mcc)	Merkel carcinoma	\
			GSM1914648 (mcc)		
			GSM1914649 (mcc)		
			GSM1914650 (mcc)		

**Notes:** A double channel chip was marked with a star sign.

Study 1 (GSE26032) was conducted in the head and neck squamous cell carcinoma(HNSC) cell line, SAS and FaDu, treated with miR-357 to explore the genes that are differently expressed using a GPL10332 platform containing 44,495 probes.

Study 2 (GSE31566) was performed in miR-375-transfected lung cancer A549, was conducted to examine changes in expression of potential target genes of miR-375 by transfection of Pre-miR-375 or Pre-miR-NC#2 (Ambion) in A549 cells containing 41,000 probes, which were then harvested at 12, 24, 48, and 96 hours after transfection.

Study 3 (GSE37119) was conducted with miR-375-transfected head and neck squamous cell carcinoma (IMC-3) and esophageal squamous cell carcinoma (TE2) by GPL10332 with 44,495 probes.

Study 4 (GSE77790) determined potential miR-375 target genes in pancreatic cancer and esophageal cancer (Panc-1 and sw1990 & TE8 and TE9). The study collected miR-375 target genes with GPL20844 and included 62,976 probes.

Study 5 (GSE47657) was conducted with miR-375-transfected head and neck squamous cell carcinoma cells (HNSC) FaDu were treated with miR-375 a GPL13607 platform containing 62,976 probes.

Study 6 (GSE58860) was performed in miR-375 transfected Colon cancer cell lines SW480 using a GPL10558 platform containing 18,766 probes.

Study 7 (GSE67742) was performed in miR-375-transfected in medullary thyroid carcinoma cells ORI by a GPL16699 platform containing 62,976 probes.

Study 8 (GSE40058) was conducted with miR-375-transfected breast cancer cells MDA-MB-231 transfected via miR-375a GPL6244 platform containing 33,297 probes.

Study 9 (GSE74213) determined potential miR-375 target genes in merkel cell carcinoma cell lines MCC26 with a GPL6480 platform includes 28,376 probes.

### Differentially down-regulated genes by Robust Rank Aggregation (RRA) analysis and predicted genes

**Fig 2:**
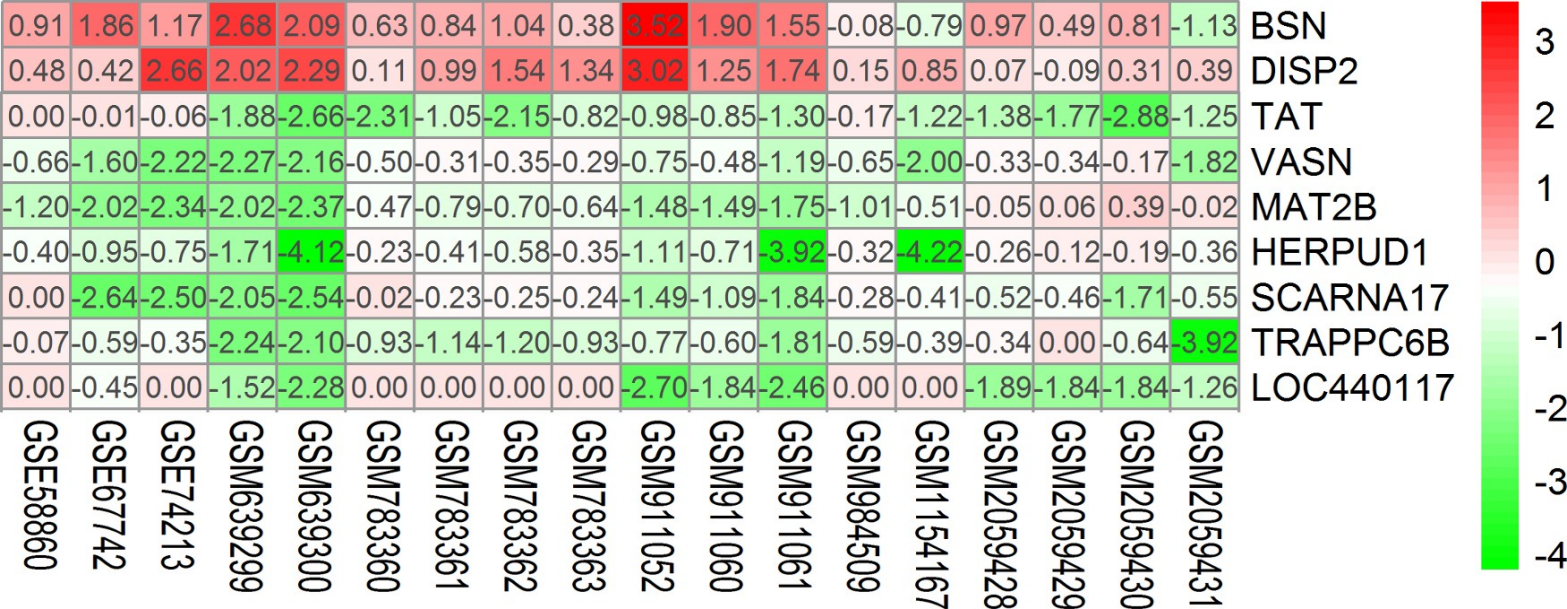
Heat map of 9 differentially expressed genes of Robust Rank Aggreg (RRA) analysis. Different color represented fold change of gene expression. Specific fold change values were written in each gene box.

**Fig 3:**
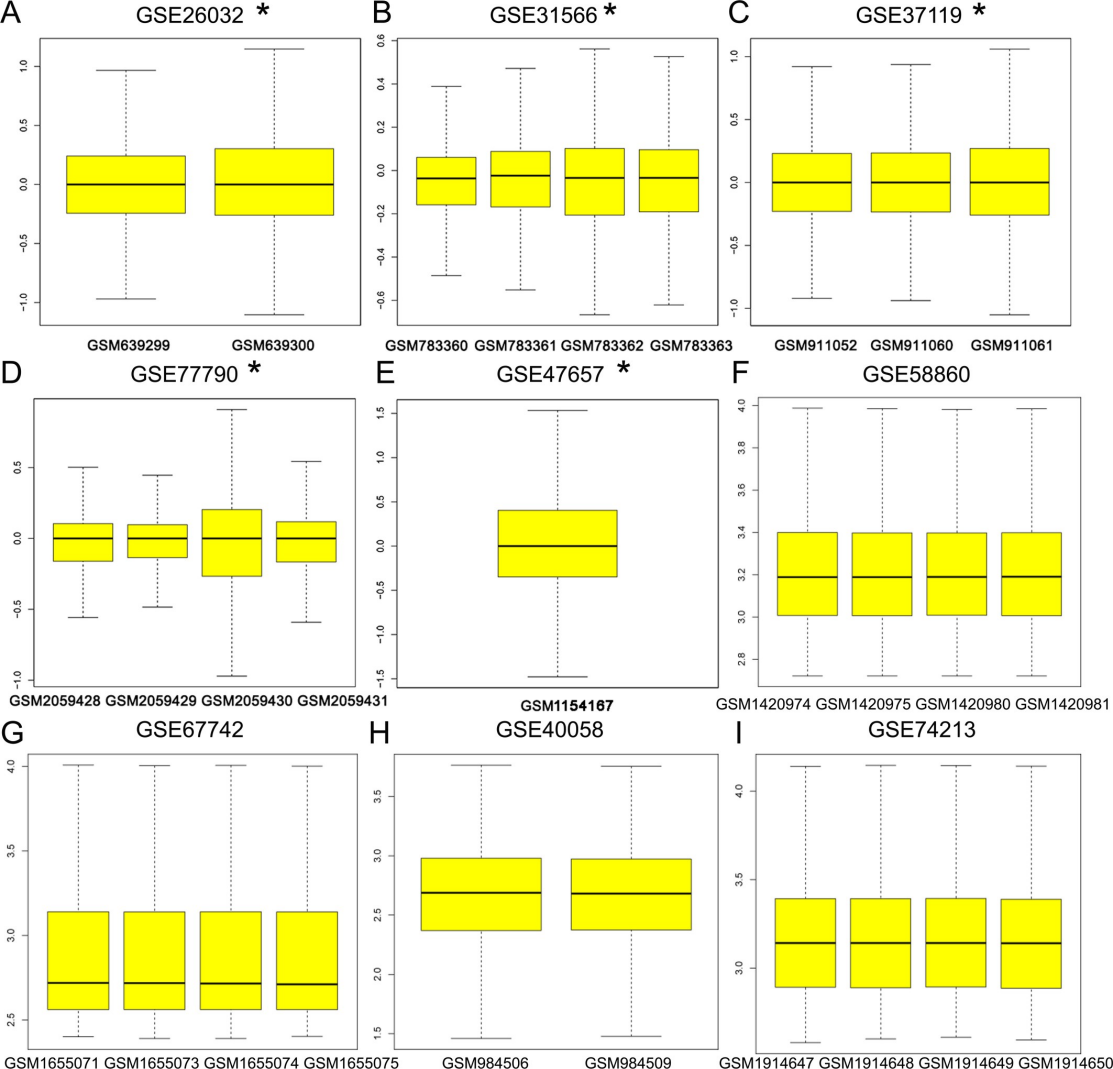
Box-plot showed normalized value of the 9 series data sets. A double channel chip was marked with a star sign.

**Fig 4:**
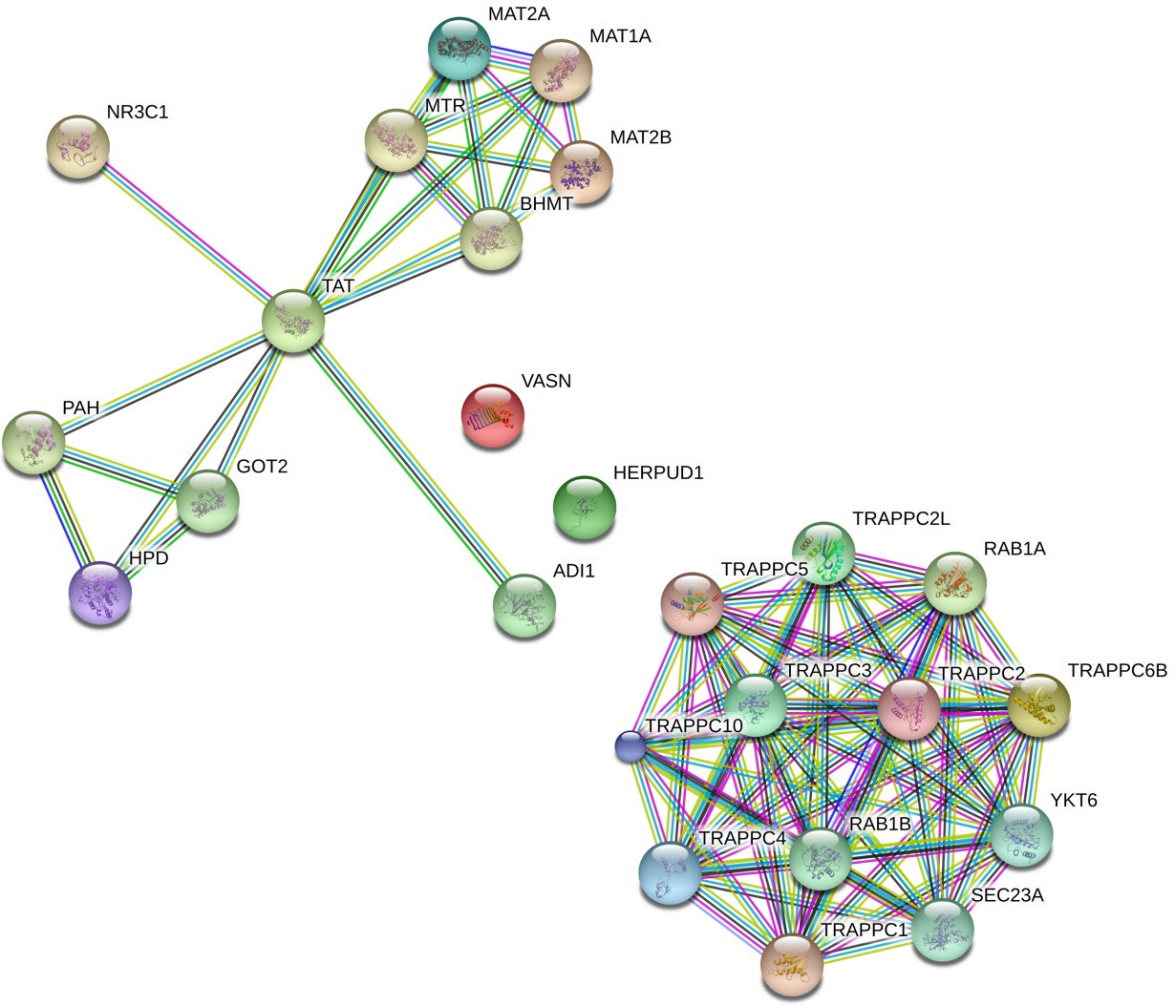
Protein protein interaction network of 25 potential targets of miR-375. Protein protein interaction network was generated by STRING online tool. High confidence (0.700) was set to the interaction score.

Nine differentially expressed genes were obtained through RRA analysis, of which 2 were up regulated and 7 down regulated (Fig 2). Five genes, TAT, VASN, MAT2B, HERPUD1 and TRAPPC6B, were obtained from the intersection of 7 down-regulated genes and predictive genes of miR-375 (Table 2). Box-plots used to show the statistical distribution of GSE data and variation between groups (Fig 3). Since there are only 5 genes, we use the Max number of interactors to show: no more than 20 interactors, to expand the number of related genes. As a result, 5 original genes and 20 extended genes were obtained. S1 Table shows that 19 of the 20 extended genes are predictive genes for miR-375. A protein interaction network was constructed using these 25 genes (Fig 4).

**Table 2:** Representation of the potential target genes gathered from RRA and miRWalk2.0.

Gene	P-value	FDR	Log2FC
TAT	6.96E-18	4.45E-13	-1.263439706
VASN	1.82E-16	1.16E-11	-1.003717538
MAT2B	4.33E-16	2.76E-11	-1.022535943
HERPUD1	1.58E-15	1.01E-10	-1.151217131
TRAPPC6B	9.55E-15	6.10E-10	-1.035148781

**Abbreviations:** FDR, false discovery rate; FC, fold change.

### The GO and KEGG function enrichment analysis of target gene

**Fig 5:**
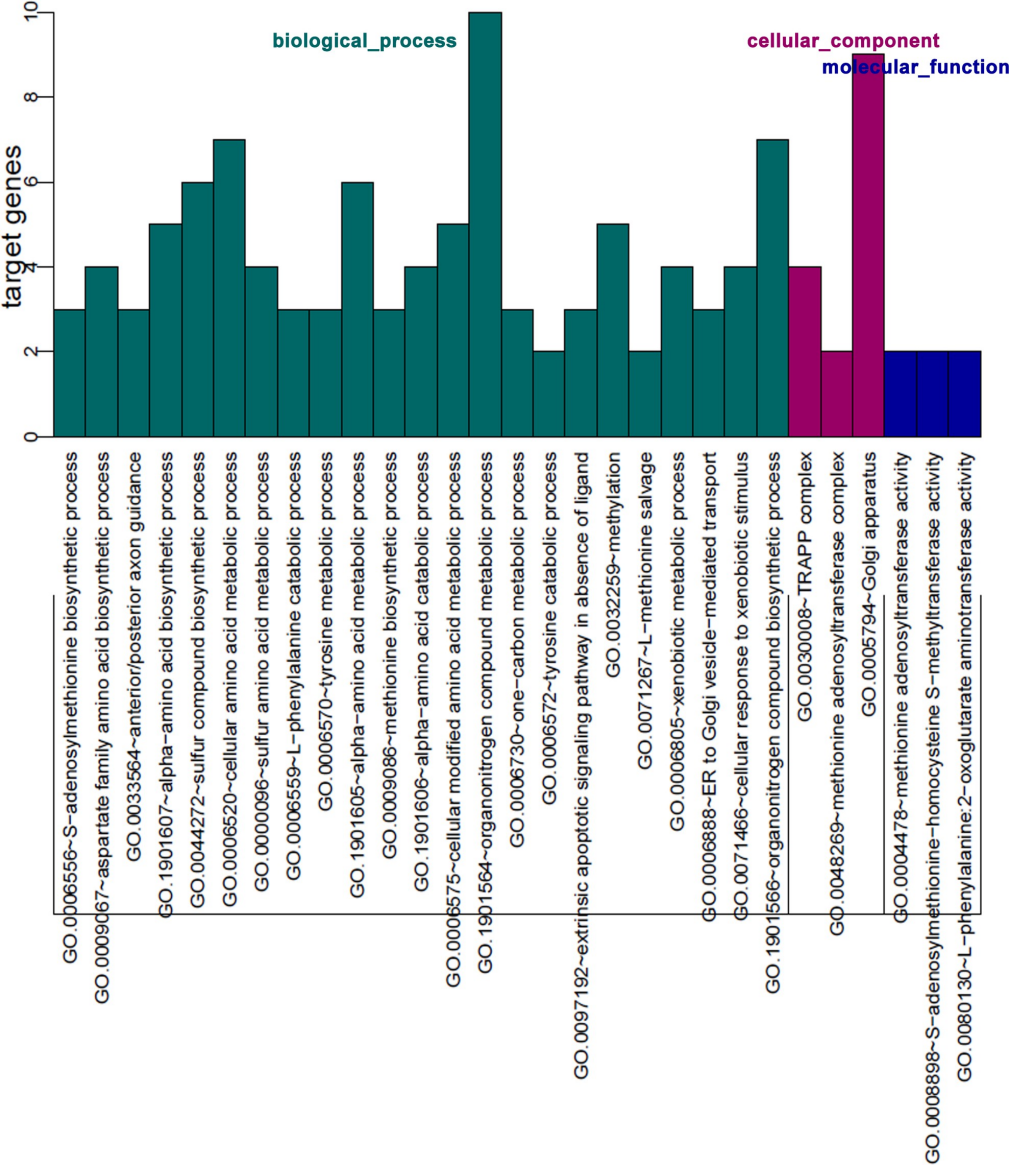
Gene Ontology (GO) annotation of 25 potential targets of miR-375. The bar plot of GO Annotation was drawn by R language. GO Annotation was generated using STRING online tool. The Y axis indicated the number of enriched genes.

In order to better explore the function of miR-375, the 25 genes were used for functional enrichment analysis of GO and KEGG. Many GO categories related to the specific biological functions are enriched in these genes with the highest FDR, such as TRAPP complex, Golgi apparatus and endoplasmic reticulum in cellular components (CC), ER to Golgi vesicle-mediated transport, Golgi vesicle transport and S-adenosylmethionine biosynthetic process in biological processes (BP) and methionine adenosyltransferase activity, S-adenosylmethionine-homocysteine and 2-oxoglutarate aminotransferase activity in molecular functions (MF) (Fig 5 and S2 Table). KEGG pathway enrichment found that these genes had regulatory effects in amino acid biosynthesis and metabolic pathway, e.g, cysteine and methionine metabolism and metabolic pathways (Table 3).

**Table 3:** KEGG analysis of 25 potential targets related to miR-375.

Pathway ID	Pathway description	Gene count	FDR
270	Cysteine and methionine metabolism	8	1.21E-14
1230	Biosynthesis of amino acids	7	2.65E-10
360	Phenylalanine metabolism	4	3.91E-07
400	Phenylalanine, tyrosine and tryptophan biosynthesis	3	1.15E-06
1100	Metabolic pathways	10	2.86E-05
350	Tyrosine metabolism	3	0.000583
130	Ubiquinone and other terpenoid-quinone biosynthesis	2	0.00263

**Abbreviations:** FDR, false discovery rate.

### MiR-375 expression levels in various cancers based on TCGA data

**Fig 6:**
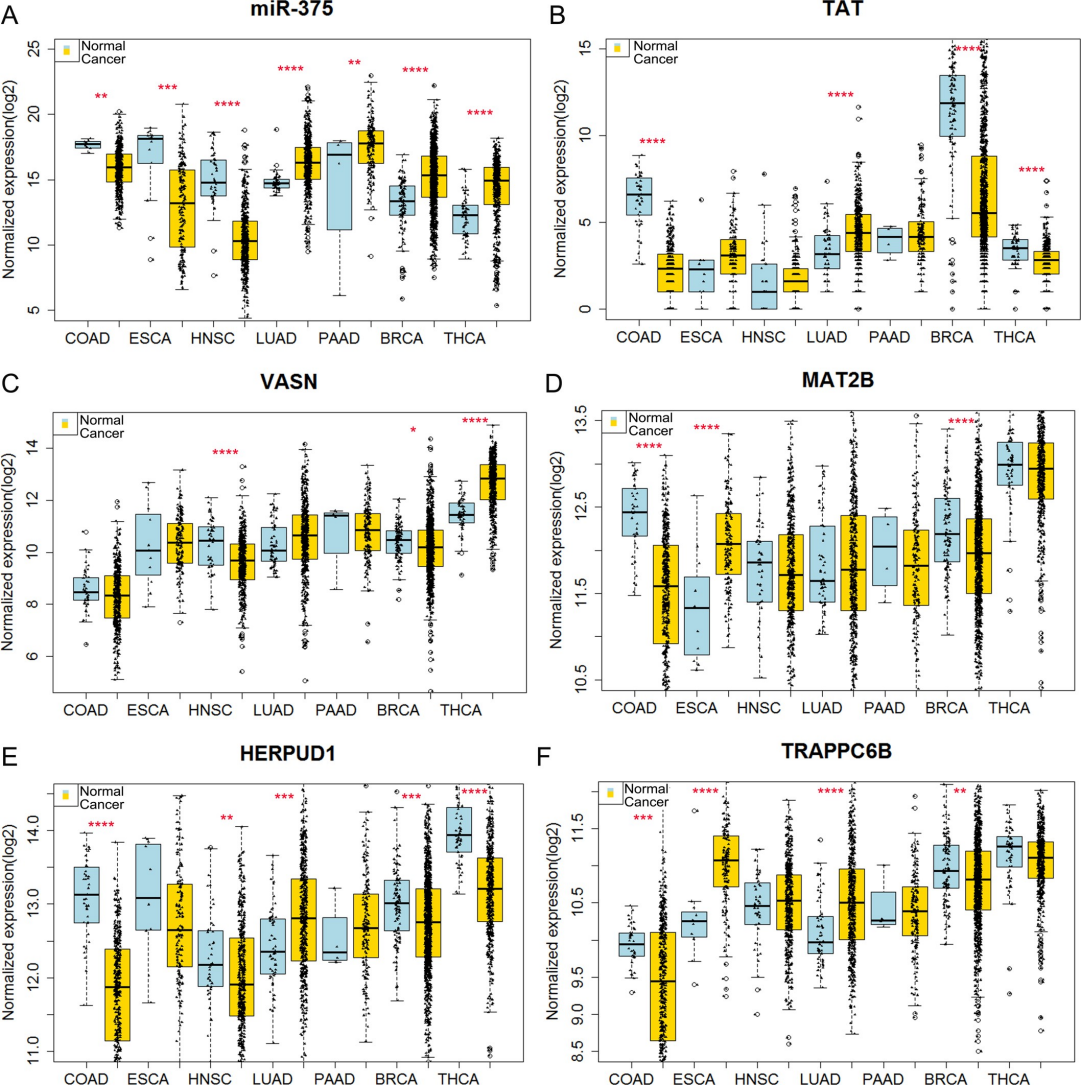
The expression levels of miR-375 as well as its target genes in various cancers from TCGA data. The boxplots were generated by boxplot of R language. (A) The expression levels of miR-375 in colon adenocarcinoma(COAD), esophageal carcinoma(ESCA), head and neck squamous cell carcinoma (HNSC), lung adenocarcinoma (LUAD), pancreatic adenocarcinoma (PAAD), breast invasive carcinoma (BRCA) and thyroid Carcinoma (THCA). (B) The expression levels of TAT in various tumors. (C) The expression levels of VASN in various tumors. (D) The expression levels of MAT2B in various tumors. (E) The expression levels of HERPUD1 in various cancers. (F) The expression levels of TPAPPC6B in various cancers. Counts data are log2 transformed. *: p< 0.05; **: p<0.01; ***: p<0.001; ****p< 0.0001.

The expression levels of miR-375 and its target genes were shown in Fig 6. miR-375 in colon adenocarcinoma (COAD), esophageal carcinoma (ESCA), head and neck squamous cell carcinoma (HNSC), lung adenocarcinoma (LUAD), pancreatic adenocarcinoma (PAAD), breast invasive carcinoma (BRCA) and thyroid carcinoma (THCA) expression. We used Pearson's correlation test to detect the correlation between miR-375 and target genes. The results are as follows:

MiR-375 shows negatively relationship with VASN, MAT2B as well as TPAPPC6B in ESCA.
MiR-375 shows negatively relationship with VASN, MAT2B as well as TPAPPC6B in HNSC.
MiR-375 shows negatively relationship with VASN, MAT2B as well as HERPUD1 in LUAD.
MiR-375 shows negatively relationship with VASN as well as MAT2B in PAAD.
MiR-375 shows negatively relationship with VASN, MAT2B as well as HERPUD1 in BRCA.
MiR-375 shows negatively relationship with TAT as well as HERPUD1 in THCA.

**Fig 7:**
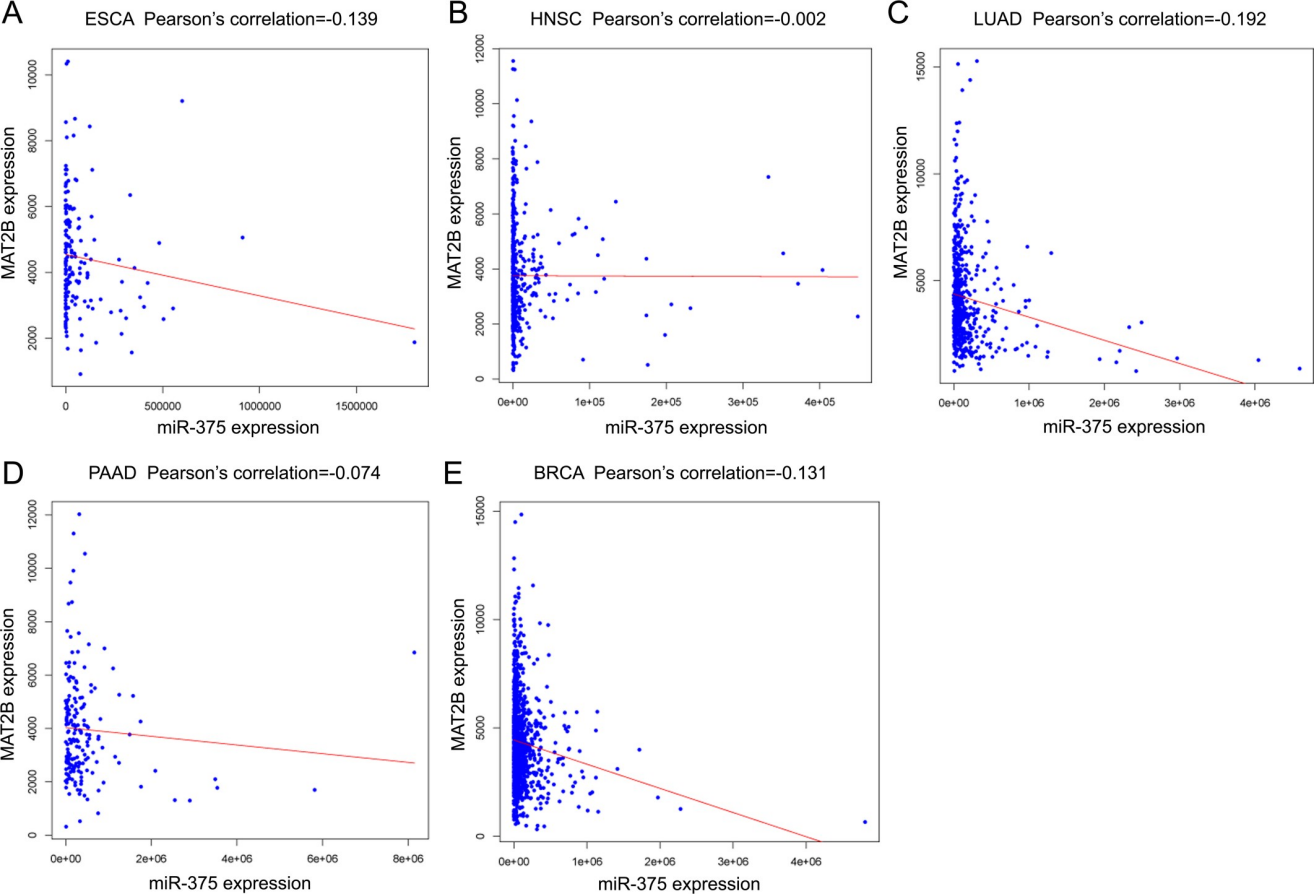
Correlations between miR-375 and MAT2B in 5 cancers. The correlation between mir-375 and MAT2B was illustrated by Pearson's correlation test. (A) esophageal carcinoma (ESCA), (B) head and neck squamous cell carcinoma (HNSC), (C) lung adenocarcinoma (LUAD), (D) pancreatic adenocarcinoma (PAAD), (E) breast invasive carcinoma (BRCA).

The negative correlation between mir-375 and MAT2B in 5 tumors was displayed in Fig 7.

## Discussion

MiRNA is a conserved small 19–24 nucleotide non-coding RNA, it adjusts gene expression in a sequence-specific pattern [4]. Most miRNA are transcribed in the nucleus through RNA polymerase II in the beginning of replication [27]. By binding to 3 'untranslated region messenger RNA (UTR), the expression of the target gene is suppressed or stopped by multiple mechanisms include enhanced conversion compression and messenger RNA degradation [28]. It is well known that they have the ability to regulate important cellular processes, for example, cell differentiation, cell cycle, proliferation and apoptosis. They can play a role as oncogenes or tumor suppressor in a variety of cancers. Recent studies have shown that nearly 50% of miRNAs is associated with tumor-related sites in the genome. They can come into play as oncogenes or tumor suppressor genes by recognizing and binding to targeted molecules, or induce mRNA degradation or posttranscriptional regulation of mRNA translation [29]. Study about the relationship between miRNAs and cancer was initially illustrated in B cells chronic lymphoblastic leukemia [30] and an increasing number of studies have been suggested the biological function of microRNA is highly related to human carcinogenesis, ovary cancer, lung cancer, breast cancer, liver and larynx cancer are no exception [31]. These studies suggest that miRNAs plays a crucial role in tumorigenesis and development [32].

Previous meta analysis confirmed under-expression of miR-375 in several cancers. A growing number of data suggests that the down-regulation of miR-375 is related to poor overall survivability in various cancers, suggesting that the expression of mir-375 is connected with OS in patients with malignant tumors. It can be used as a useful biomarker for clinical prognosis, which has been confirmed by recent meta analysis [33]. This meta analysis includes esophageal cancer (8 studies), non-small cell lung cancer (NSCLC 3 studies), glioma, breast cancer, gastric cancer, head and neck squamous cell carcinoma (HNSCC), pancreatic ductal adenocarcinoma (PDAC) 16 studies in total, the results confirmed that the down-regulation of miR-375 may predict these 7 kinds of cancer overall survival rate, which are conform to our consequence of miR-375 expression levels in various cancers based on TCGA data including colon adenocarcinoma (COAD), esophageal carcinoma (ESCA), head and neck squamous cell carcinoma (HNSC).

In our study, we gained insight into miR-375 and investigated its potential signaling pathways in various cancers with different cell lines by mining the microarray data after miR-375 mimic transfection. These cell lines contained head and neck squamous cell carcinoma cells SAS and FaDu (GSE26032), lung cancer cells A549 (GSE31566), head and neck squamous cell carcinoma cells IMC-3 and esophageal squamous cell carcinoma cells T.Tn and TE2 (GSE37119), pancreatic cancer cells Panc-1 and sw1990 and esophageal cancer cells TE8 and TE9 (GSE77790), head and neck squamous cell carcinoma cells FaDu (GSE47657), colon cancer cell lines SW480 (GSE58860), medullary thyroid carcinoma cells ORI (GSE67742), breast cancer cells MDA-MB-231 (GSE40058), merkel cell carcinoma cell lines MCC26 (GSE74213). In addition, the target genes of miR-375 have been identified (TAT, VASN, MAT2B, HERPUD1 and TPAPPC6B).

Totally, 5 key target genes and 20 extended genes were found that were intensely enriched in amino acid biosynthetic and metabolic process from biological process pathway (GO). These key pathways assist in explaining the miR-375 mechanism, which was significantly down-regulated in the migration and invasion of multiple cancers and the cysteine and methionine metabolism, biosynthesis of amino acids, phenylalanine metabolism, as well as phenylalanine, tyrosine and tryptophan biosynthesis from KEGG pathway analysis. These correlated pathways assist in illustrating the miR-375 mechanism, which was significantly down-regulated in the migration and invasion of multiple cancers. We are interested in the expression of miR-375 in colon adenocarcinoma (COAD), esophageal carcinoma (ESCA), head and neck squamous cell carcinoma (HNSC), lung adenocarcinoma (LUAD), pancreatic adenocarcinoma (PAAD), breast invasive carcinoma (BRCA) and thyroid carcinoma (THCA). Because they were involved in in vitro cell studies of the microarray data above, as expected, the reduction in miR-375 levels is well documented in some of these cancers.

In our study, 5 key target genes (TAT, VASN, MAT2B, HERPUD1 and TRAPPC6B) were discovered and their several biological functions have been found in previous researches. The role of TAT in viral transcription has been well studied, especially in HIV [34]. TAT binds to activate the transcription complex and assembles it onto the viral promoter, causing a dramatic increase in viral transcript. TAT can be released by infected cells, inducing physiological changes in normally uninfected cells such as neurons and endothelial cells. In addition to promoting viral transcription, TAT regulates the expression of cellular genes, regulates key pathways and mechanisms to produce an environment conducive to the generation and transmission of HIV, and the up-regulated genes of TAT appear to play a positive role in the activation of T cells and the promotion of virus replication and transmission [35]. The extracellular domain of VASN is composed of leucine-rich tandem repeats, epidermal growth factor (EGF) -like motifs and fibronectin type iii motifs. Expression analysis indicated that VASN was mainly expressed in vascular smooth muscle cells, and the down-regulation of vasorin expression was an important cause of acute vascular injury. Its binding partners are the transforming growth factor (TGF)- ca and VASN can directly attenuates TGF- could signaling in vitro, which is because VASN is directly bound to TGF- but negatively regulates TGF- signaling in the vascular wall, and at least to some extent, inhibits the formation of neointima by negative regulation [36]. In other studies,VASN was further confirmed to be able to promote cell proliferation and migration, and to promote cell proliferation and migration and inhibit cell apoptosis. As a membrane protein and/or free protein,VASN may be an effective target for biological treatment of liver cancer and a potential biomarker for the diagnosis of liver cancer [37]. Extracellular domain structure can be tumor necrosis factor—invertase (conventional) pyrolysis and then directly with shift growth factor beta (TGF—beta) and inhibition of TGF—beta signaling, thus we speculated that VASN may be mediator between tumor progression and angiogenesis, and it also can promote cancer cell proliferation and migration, which is a transmembrane protein VASN regulating tumor metastasis through the new way. The study suggested that vasn-positive exosomes provide a signal of metastasis from primary tumor cells to surrounding cells [38]. As for MAT2B, it has been demonstrated to inhibit cell growth, colony formation and induction of apoptosis of A375 and mel-rm cell lines in vitro, affect the expression of BCL2 and XAF1 proteins, and prolong the growth of transplanted tumor in vivo. MAT2B is crucial in the proliferation and tumorigenicity of melanoma cells [39]. Previous studies have found that MAT2B and GIT1 interact and are overexpressed in most human liver cancer and colon cancer specimens. Both MAT2B variants (overexpressed V1, V2) interact with GIT1. MAT2B and GIT1 form a scaffold that activates MEK and ERK, promoting growth and tumorigenesis [40,41]. HERPUD1 Herp said again, it is a kind of 54 kDa across the endoplasmic reticulum (ER) membrane protein, participate in ER related protein degradation pathway, involved in ER steady-state its levels rise sharply in ER stress, HEPDU1 by cutting inositol 1, 4–3 phosphoric acid phosphate receptor protect the cell apoptosis induced by oxidative stress, Herp mediated by promoting proteasome ER—resident Ca2 + release channel degradation to maintain ER steady state [42,43]. HERPUD1 plays a role in protein response (UPR) and endoplasmic reticulum (ER) related protein degradation (ERAD) [44,45]. Transport protein particle (TRAPP) is a series of related protein complexes acting at a specific stage of cell organelle traffic. They share a core subunit that can be activated by the guanine nucleotide exchange factor (GEF) activity to activate GTPase Rab1 and distinguished by "dependent" subunits that give each complex different functions. These subunits are widely expressed, so mutations in the TRAPP subunit could be fatal mutations in the embryonic stage. Since its discovery, however, many subunits have been found to have mutated in several different human diseases, suggesting that some of those subunits may have cell-or tissue-specific functions [46]. TRAPP contains a series of protein complexes that control membrane transport from the endoplasmic reticulum (ER) to the golgi and plasma membranes. TRAPP I regulates vesicle transport from ER to early golgi body,TRAPP II controls transport in golgi body, and autophagy requires the participation of TRAPP III [42].

However, there are still more unknown miR-375 targets for cancer. For example, our previous study found that miR-375 expression level was low in lung cancer, including squamous cell carcinoma and adenocarcinoma and small cell lung cancer [47,48]. The expression level of miR-375 was inhibited in DU145 and PC-3 cell lines and clinical samples, and cell proliferation and invasion were inhibited by targeting Sec23 homolog A and yes-associated protein 1(SEC23A and YAP1), while inducing apoptosis and ectopic recovery of cell cycle arrest [49]. MiR-375 is also expressed in other squamous cell carcinomas (SCCs), including head and neck, oral, esophageal, pancreatic islets and larynx SCCs [1–3]. MiR-375 can directly target the matrix metalloproteinase 13 (MMP13), insulin-like growth factor-1 receptor (IGF-1R), zinc finger protein 36 ring finger protein 2 (ZFP36L2), Y box binding protein 1 (YBX1), and inositol-trisphosphate 3-kinase B (ITPKB) to inhibit SCC cell proliferation and migration [1–3,33,50]. In thyroid medullary carcinoma, miR-375 was also down-regulated and associated with metastasis possibly by targeting SEC23A [51]. MiR-375 was down-regulated in colorectal cancer (CRC) by inhibiting Bcl-2 pathway control of CRC tumor cells [52]. Some of the target genes were also confirmed including Frizzled 8 (FZD8) [53], aromatic hydrocarbon receptor (AhR) [54], ERBB2 (HER2) [55] and Janus kinase (JAK2) [56].

In addition, more targets are confirmed in other diseases. MiR-375 has been reported to inhibit diabetes by targeting the expression of mRNA targets of miR-375 such as Gephyrin (GPHN), Insulin induced gene 2 (INSIG2), Myotrophin (MTPN) and Eukaryotic translation elongation factor 1 (Eef1e1) [57]. In her2-positive breast cancer [58] and oral squamous cell carcinoma[1], silencing of miR-375 leads to up-regulation of IGF1R, which was found to be a direct target of miR-375. MiR-375 was found to be the target of Ubiquitin-protein ligase E3A (UBE3A) in cervical cancer[59,60], and the improvement of radio sensitivity in HR-HPV (+) cervical cancer is highly correlated with miR-375-UBE3A axis.

Among these confirmed articles, miR-375 showed results that closely resembled our findings, suggesting that miR-375 has a number of common goals in the cancers involved, including the seven cancers we studied (COAD, ESCA, HNSC, LUAD, PAAD, BRCA and THCA).

In order to find out the role of target genes that have been identified in this study in other miRNA regulation, we further sifted through studies already published on pubmed to further explore the level of mRNA expression in these genes (TAT, VASN, MAT2B, HERPUD1 and TPAPPC6B). It was found that most of the miR-375 levels were negatively related to different cancers. The roles of these genes in invasiveness and metastasis have been confirmed.

VASN acts as an essential part in the growth and migration of HepG 2 cells [37] and increases apoptosis through down-regulation of VASN expression. Down regulation of miR-145 and miR-146a is a significant mechanism causing VASN high expression. Targeting small nucleotide siRNA or aptamer of VASN may be a promising drug therapy for HCC.

Low expression of miR-9-3p leads to a high level of Herpud 1, which prevents glioma cell apoptosis [61]. The function of Herpud1 is not totally figured out, but there is more and more evidence that it plays an important role in ER-membrane-associated protein degradation (ERAD) by transferring ubiquitin protein from endoplasmic reticulum (ER) to proteasome degradation [62]. In U251 cells with high level of miR-9-3p, the expression of HERPUD1 increased while downtrend was performed in the mRNA and protein levels of HERPUD1. More apoptotic cells were displayed after H2O2 induced by the miR-9-3p simulated U251 cells. A similar pattern was discovered in herpud1-knockdown cells. Thus, these results suggest that Herpud1 can protect glioma cells from oxidative stress. Glioma may form a strategy of antioxidant stress by down-regulating miR-9-3p, thereby upregulating its target gene HERPUD1. Recent studies have also identified Herpud1 as a cytokine of antioxidant stress [43].

Studies by Lemkul JA et al suggest that TAT has vital effectin hepatitis and liver cancer as well as tyrosinemia type II [63]. Simultaneous luciferase reporter assays ensured that miR-133a targets TAT, and that miR-133a gets command of the contractility of diabetic hearts by targeting TAT, regulating neurobiosis, and thereby regulating β-AR and cardiac function [64].

Over-expression of miR-34a or miR-34b lead to the low expression level of MAT2B, mainly in the protein expression level of CWR22Rv-1 and MIA PaCa-2 cells [65]. Using microRNA microarray, Lo TF et al [66] found that after berberine treatment, the expression of miR-21-3p (previously named miR-21) up-regulated in HepG2 human hepatoma cell line. MiR-21-3p decreased the expression of MAT2B directly through direct targeting 3’UTRs. Furthermore, over-expression of miR-21-3p augmented intracellular SAM content, restrained cell growth and migration as well as apoptosis of HepG2 cells, indicating its therapeutic potential in hepatocellular carcinoma. MAT2B can induce the growth and survival of cancer cells, enhance tumor migration and play a carcinogenic role. The increased expression of MAT2B happens in human liver, colon, gastric, breast, pancreas and prostate cancer [67]. In addition to interacting with MAT II, the mutations of MAT2B can interact with other else proteins, for example, including HuR [68].

## Conclusions

In conclusion, miR-375 overexpressed cell lines from pan-cancers were innovatively converged, and its common target genes were extracted. This research provides a new perspective for exploring the molecular mechanism of miR-375 in human tumors. However, the accurate molecular mechanism of miR-375 was demanded to be validated in further investigations.

## Supporting information

S1 Table 19 of the 20 extended genes are predictive genes for miR-375. (DOCX)

S2 Table Gene Ontology (GO) annotation of 25 potential targets of miR-375. (DOCX)
