# Supplementary material for: The biological functions of target genes in pan-cancers and cell lines were predicted by miR-375 microarray data from GEO database and bioinformatics
Source: PLoS One. 2018 Oct 31;13(10):e0206689. doi: 10.1371/journal.pone.0206689 (PMC6209324; doi:10.1371/journal.pone.0206689)
Supplement: S1 Table — (DOCX) [file pone.0206689.s001.docx]

**S1 Table. 19 of the 20 extended genes are predictive genes for miR-375**

| **Gene** | **EntrezID** | **miRWalk** | **Microt4** | **miRanda** | **mirbridge** | **miRDB** | **miRMap** | **miRNAMap** | | **Pictar2** | | **PITA** | | **RNA22** | | **RNAhybrid** | | **Targetscan** | | **SUM** | |
| --- | --- | --- | --- | --- | --- | --- | --- | --- | --- | --- | --- | --- | --- | --- | --- | --- | --- | --- | --- | --- | --- |
| MTR | 4548 | 1 | 1 | 0 | 1 | 0 | 1 | 0 | 0 | | 1 | | 1 | | 1 | | 1 | | 8 | |  |
| BHMT | 635 | 1 | 1 | 0 | 0 | 0 | 1 | 0 | 0 | | 1 | | 1 | | 1 | | 1 | | 7 | |  |
| SEC23A | 10484 | 1 | 1 | 0 | 0 | 0 | 1 | 1 | 0 | | 0 | | 1 | | 1 | | 1 | | 7 | |  |
| YKT6 | 10652 | 0 | 0 | 0 | 0 | 0 | 1 | 0 | 0 | | 1 | | 1 | | 1 | | 0 | | 4 | |  |
| RAB1A | 5861 | 0 | 1 | 0 | 0 | 0 | 0 | 0 | 0 | | 0 | | 1 | | 1 | | 0 | | 3 | |  |
| RAB1B | 81876 | 0 | 0 | 0 | 0 | 0 | 0 | 0 | 0 | | 0 | | 1 | | 1 | | 0 | | 2 | |  |
| NR3C1 | 2908 | 0 | 1 | 0 | 0 | 0 | 0 | 0 | 0 | | 0 | | 0 | | 1 | | 0 | | 2 | |  |
| GOT2 | 2806 | 0 | 0 | 0 | 0 | 0 | 1 | 0 | 0 | | 0 | | 0 | | 1 | | 0 | | 2 | |  |
| TRAPPC10 | 7109 | 0 | 0 | 0 | 0 | 0 | 0 | 0 | 0 | | 0 | | 1 | | 1 | | 0 | | 2 | |  |
| MAT1A | 4143 | 0 | 0 | 0 | 0 | 0 | 0 | 0 | 0 | | 0 | | 1 | | 1 | | 0 | | 2 | |  |
| ADI1 | 55256 | 0 | 0 | 0 | 0 | 0 | 0 | 0 | 0 | | 0 | | 1 | | 1 | | 0 | | 2 | |  |
| PAH | 5053 | 0 | 0 | 0 | 0 | 0 | 0 | 0 | 0 | | 0 | | 1 | | 1 | | 0 | | 2 | |  |
| TRAPPC1 | 58485 | 0 | 0 | 0 | 0 | 0 | 0 | 0 | 0 | | 0 | | 1 | | 1 | | 0 | | 2 | |  |
| TRAPPC2 | 6399 | 0 | 0 | 0 | 0 | 0 | 1 | 0 | 0 | | 0 | | 0 | | 1 | | 0 | | 2 | |  |
| TRAPPC2L | 51693 | 0 | 0 | 0 | 0 | 0 | 0 | 0 | 0 | | 0 | | 0 | | 1 | | 0 | | 1 | |  |
| TRAPPC3 | 27095 | 0 | 0 | 0 | 0 | 0 | 0 | 0 | 0 | | 0 | | 0 | | 1 | | 0 | | 1 | |  |
| TRAPPC4 | 51399 | 0 | 1 | 0 | 0 | 0 | 0 | 0 | 0 | | 0 | | 0 | | 0 | | 0 | | 1 | |  |
| TRAPPC5 | 126003 | 0 | 0 | 0 | 0 | 0 | 0 | 0 | 0 | | 0 | | 1 | | 0 | | 0 | | 1 | |  |
| MAT2A | 4144 | 0 | 0 | 0 | 0 | 0 | 0 | 0 | 0 | | 0 | | 0 | | 1 | | 0 | | 1 | |  |
| HPD | 3242 | 0 | 0 | 0 | 0 | 0 | 0 | 0 | 0 | | 0 | | 0 | | 0 | | 0 | | 0 | |  |
