# Supplementary material for: The biological functions of target genes in pan-cancers and cell lines were predicted by miR-375 microarray data from GEO database and bioinformatics
Source: PLoS One. 2018 Oct 31;13(10):e0206689. doi: 10.1371/journal.pone.0206689 (PMC6209324; doi:10.1371/journal.pone.0206689)
Supplement: S2 Table — (DOCX) [file pone.0206689.s002.docx]

**S2 Table. Gene Ontology (GO) annotation of 25 potential targets of miR-375**

| **Pathway ID** | **pathway description** | **Gene count** | **FDR** |
| --- | --- | --- | --- |
| **Biological Process** | |  |  |
| GO.0006888 | ER to Golgi vesicle-mediated transport | 7 | 9.34E-09 |
| GO.0048193 | Golgi vesicle transport | 8 | 1.24E-06 |
| GO.0006556 | S-adenosylmethionine biosynthetic process | 3 | 7.41E-06 |
| GO.0009067 | aspartate family amino acid biosynthetic process | 4 | 1.41E-05 |
| GO.0016192 | vesicle-mediated transport | 11 | 3.48E-05 |
| GO.1901607 | alpha-amino acid biosynthetic process | 5 | 3.48E-05 |
| GO.0016482 | cytoplasmic transport | 9 | 8.48E-05 |
| GO.0044272 | sulfur compound biosynthetic process | 6 | 8.48E-05 |
| GO.0006520 | cellular amino acid metabolic process | 7 | 0.000109 |
| GO.0000096 | sulfur amino acid metabolic process | 4 | 0.000191 |
| GO.0006559 | L-phenylalanine catabolic process | 3 | 0.000204 |
| GO.0006570 | tyrosine metabolic process | 3 | 0.000204 |
| GO.1901605 | alpha-amino acid metabolic process | 6 | 0.000213 |
| GO.0009086 | methionine biosynthetic process | 3 | 0.000371 |
| GO.1902582 | single-organism intracellular transport | 9 | 0.00134 |
| GO.1901606 | alpha-amino acid catabolic process | 4 | 0.00156 |
| GO.0006575 | cellular modified amino acid metabolic process | 5 | 0.00178 |
| GO.1901564 | organonitrogen compound metabolic process | 10 | 0.0022 |
| GO.0006730 | one-carbon metabolic process | 3 | 0.00423 |
| GO.0006572 | tyrosine catabolic process | 2 | 0.0068 |
| GO.0032259 | methylation | 5 | 0.00761 |
| GO.0044710 | single-organism metabolic process | 14 | 0.0111 |
| GO.0071267 | L-methionine salvage | 2 | 0.0149 |
| GO.0006805 | xenobiotic metabolic process | 4 | 0.0164 |
| GO.0044281 | small molecule metabolic process | 10 | 0.0172 |
| GO.0071466 | cellular response to xenobiotic stimulus | 4 | 0.0172 |
| GO.0046907 | intracellular transport | 8 | 0.0186 |
| GO.1901566 | organonitrogen compound biosynthetic process | 7 | 0.0189 |
| GO.0044248 | cellular catabolic process | 8 | 0.0327 |
| GO.0006732 | coenzyme metabolic process | 4 | 0.0415 |
| GO.1903020 | positive regulation of glycoprotein metabolic process | 2 | 0.0492 |
| **Cellular Componment** | |  |  |
| GO.0030008 | TRAPP complex | 4 | 1.42E-08 |
| GO.0005794 | Golgi apparatus | 13 | 5.54E-07 |
| GO.0005783 | endoplasmic reticulum | 12 | 2.07E-05 |
| GO.0048269 | methionine adenosyltransferase complex | 2 | 0.000586 |
| GO.0005829 | cytosol | 13 | 0.00419 |
| GO.0012505 | endomembrane system | 13 | 0.00924 |
| GO.0044444 | cytoplasmic part | 18 | 0.0157 |
| **Molecular Function** | |  |  |
| GO.0004478 | methionine adenosyltransferase activity | 2 | 0.00589 |
| GO.0008898 | S-adenosylmethionine-homocysteine S-methyltransferase activity | 2 | 0.00589 |
| GO.0080130 | L-phenylalanine:2-oxoglutarate aminotransferase activity | 2 | 0.00589 |

**Abbreviations:** FDR, false discovery rate.
